# B Cells and Functional Antibody Responses to Combat Influenza

**DOI:** 10.3389/fimmu.2015.00336

**Published:** 2015-06-30

**Authors:** Giuseppe Lofano, Arun Kumar, Oretta Finco, Giuseppe Del Giudice, Sylvie Bertholet

**Affiliations:** ^1^Research Center, Novartis Vaccines and Diagnostics S.r.l. (a GSK Company), Siena, Italy; ^2^Dipartimento di Biologia e Biotecnologie “Charles Darwin”, Università degli Studi di Roma “La Sapienza”, Rome, Italy

**Keywords:** influenza, hemagglutinin, functional antibody responses, universal influenza vaccine, neutralizing antibodies, vaccination strategies

## Abstract

Vaccination against influenza is the most effective way to protect the population. Current vaccines provide protection by stimulating functional B- and T-cell responses; however, they are poorly immunogenic in particular segments of the population and need to be reformulated almost every year due to the genetic instability of the virus. Next-generation influenza vaccines should be designed to induce cross-reactivity, confer protection against pandemic outbreaks, and promote long-lasting immune responses among individuals at higher risk of infection. Multiple strategies are being developed for the induction of broad functional humoral immunity, including the use of adjuvants, heterologous prime-boost strategies, and epitope-based antigen design. The basic approach is to mimic natural responses to influenza virus infection by promoting cross-reactive neutralizing antibodies that directly prevent the infection. This review provides an overview of the mechanisms underlying humoral responses to influenza vaccination or natural infection, and discusses promising strategies to control influenza virus.

## Introduction

Influenza virus alone causes over 40,000 deaths every year in the United States, and even more during pandemics, like in 2009 with pandemic A/California/07/09 H1N1 virus strain (1, 2). Influenza viruses contain eight single stranded RNA segments and are classified in three different types (A, B, and C), on the basis of major antigenic differences; only influenza A and B are responsible for annual human epidemics. All influenza virus subtypes circulating in non-human species have the potential to infect humans, and transmissions from animals to humans may occur, albeit rarely, with dramatic scenarios for the public health; this was the case of the avian H5N1 strain that appeared for the first time in human in 1998 and re-appeared in 2004–2005 with a mortality rate of 50% among infected patients and thousands of deaths are reported until today (3, 4). Treatment of influenza infections is a major challenge for clinics and public health institutions because available antiviral drugs are often ineffective due to antigenic mutations or are given too late after infection (5). The most effective intervention that we have today to combat influenza is the vaccination that reduces virus infection and spreading, even if some levels of morbidity and mortality remain due to the suboptimal efficacy of the current vaccines and mismatch between the vaccine and the circulating virus strain.

Most of the current seasonal influenza vaccines are produced with live attenuated or inactivated (split or subunit) virus and both types of vaccines reduce virus infectivity and restrict viral replication by inducing functional antibodies against the virus. The antibodies generated against the virus represent the primary correlate of immunity, whereas cell-mediated immunity can contribute to reduce the clinical symptoms (6). Although existing vaccines confer acceptable levels of protection in the general population, they are suboptimal, and are associated with some important limitations: (i) antigen composition needs to be updated every year in order to match the new seasonal circulating viruses, (ii) mismatch between the vaccine and the circulating virus can always happen, and (iii) people with a reduced ability to mount an immune response, infants, the elderly, and pregnant women respond suboptimally to these vaccines, requiring a tailored vaccine formulations (7–11).

Current influenza vaccines consist of three different virus strains: two influenza A strains (usually H1N1 and H3N2) and one influenza B strain. More recently, quadrivalent influenza vaccines have been developed, which are composed of influenza B strains of both lineages (12). Unfortunately, influenza strains acquire mutations every 1–3 years in their genome segments expressing the antibody-binding regions, a process named antigenic drift, and give raise to new circulating strains. Antigenic drift represents the principal immune evasion mechanism of influenza virus and has two major consequences: first, the humoral immunity developed in response to previous infections/vaccinations is usually non-fully effective against the new emerging strains, and second, manufacturers need to update the vaccine every year with increasing costs and risks of delays in the release of the lots. The virus can also undergo major antigenic changes in his hemagglutinin (HA) and neuraminidase (NA), referred to as antigenic shift, which consists of an ample reassortment of viral gene segments between different viruses of human or zoonotic origin, leading to the emergence of totally new and potentially dangerous virus strains, as happened during the pandemics of the last century and more recently in 2009 with the H1N1 virus of swine origin (13).

In this review, we summarize the mechanisms eliciting humoral responses against influenza infection or vaccination, and discuss the approaches that are today under evaluation to develop broadly protective and, hopefully, universal vaccines against influenza.

## Learning from Antibody Responses Against Influenza

**Figure 1:**
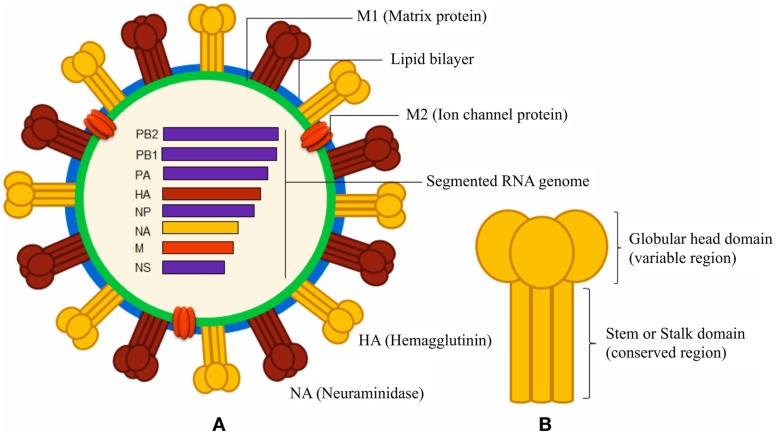
**Schematic diagrams of influenza A virus and surface hemagglutinin protein**. **(A)** The segmented negative-sense RNA genome of influenza A virus encodes three envelope proteins (hemagglutinin, neuraminidase, and ion channel M2 protein), and internal nucleoprotein (NP), polymerases (PA, PB1, and PB2), matrix protein 1 (M1), and non-structural proteins (NS). The lipid bilayer is derived from host cell membrane. **(B)** The cylindrical HA is a homo-trimeric protein consisting of a variable globular head and a conserved stem domain.

Immune responses, generated against influenza by vaccination and by natural infection, consist of neutralizing and non-neutralizing antibodies. Non-neutralizing antibodies make the most part of the antibody pool generated during the immune response, but only a small fraction is functional and participates in the clearance of infected cells through interaction with other immune cells. On the other hand, neutralizing antibodies specifically bind epitopes crucial for viral function and are extremely important to confer immunity. Most of the neutralizing antibodies recognize surface proteins of the virus, in particular, the trimeric HA, which is critical during the process of cell invasion. The overall structure of HA can be segmented in a globular head and a stem region (Figure 1). The globular head is responsible for the sialic acid-dependent binding on the extracellular surface of target cells, and allows for a conformational change of the protein for membrane fusion. Neutralizing antibodies against HA interfere during both steps of the process, in particular, they bind to the sialic acid-binding site (or in close proximity) of the globular head, thus preventing attachment of the virus to the cells. Antibodies against the stem region may restrict the conformational changes required for the membrane cell fusion. Although both kinds of antibodies are functional, only those against the stem region can have the intrinsic ability to confer broad protection against different influenza strains because this region is much less susceptible to antigenic changes as compared to the globular head (Table 1). Unfortunately, stem-specific neutralizing antibodies are rare and difficult to induce because vaccination with the seasonal vaccine formulations typically skews the specificity of B cell responses toward non-neutralizing epitopes of the stem region or, depending on the formulation of the vaccines, toward immunodominant epitopes of the HA globular head (14).

**Table 1:** **Target site of important cross-reactive neutralizing antibodies on HA stem domain**.

Neutralizing antibodies	Epitope location on HA stem	Breadth	Reference
CR6261	Helical region in the membrane-proximal stem of HA1 and HA2	A/H1, H2, H5, H6, H8, H9	(15–22)
CR9114	F subdomain	A/H1, H2, H3, H4, H5, H6, H7, H8, H9, H10, and influenza B viruses	(16, 22)
F10	Helical region in membrane-proximal stem	A/H1, H2, H5, H6, H8, H9	(21–25)
CR8020	Base of stem in close proximity to the viral membrane	A/H3, H7, H10	(26, 27)
C179	Amino acid sequences 318–322 and 47–58 of HA1and HA2, respectively	A/H1, H2, H5, H6, H9	(28, 29)

Natural responses against influenza elicit also non-neutralizing antibodies, which are specific not only to HA and NA (30) but also against M1, M2, and NP proteins (Figure 1). Such non-neutralizing antibodies, typically, can promote the clearance of the virus, relying on their Fc portion after the interaction of the variable-region of the antibody with its epitope. Several cell populations, including phagocytic cells and natural killer cells, express Fc receptors (FcRs) and may mediate the clearance of virions or virus-infected cells (31, 32). Natural killer cells express FcRs and may participate in the killing of the virus-infected cells by a mechanism called antibody-dependent cell-mediated cytotoxicity (ADCC) (33); ADCC has been observed to participate in the control of the H1N1 influenza virus infection in macaques, and a mix of intravenous antibodies that may mediate ADCC has been suggested as therapeutic for humans (34, 35). Also, the complement system may participate in clearing the virus by a mechanism called complement-dependent cytotoxicity (CDC) involving influenza-specific antibodies; in particular, IgG antibodies that bind to target surface proteins may activate complement factors in the host serum that ultimately puncture the lipid membrane of pathogen or infected host cells. Two studies have clearly shown that C3, a critical component of the complement system, may participate in reducing viral titers and in clearing the virus with a mechanism that involves M2-specific antibodies of the IgG1 or IgG2a subclasses (36, 37).

## B Cell Responses to Influenza

Most of the responding B cells after influenza vaccination or infection are specific to HA, and they are difficult to isolate and characterize, especially by flow cytometry (38), because of the binding of HA to any sialic acid residue on the host cell. Taking advantage of *ex vivo* ELISpot assay, several studies have shown that adults or older children possess low but consistent base line levels of influenza-specific IgG memory B cells, in the range of 0.1–0.6% of the total IgG memory B cells (39). Those cells respond to further antigen encounter by quickly differentiating in antibody-secreting cells, they mostly produce isotype switched antibodies and show high frequencies of mutation in their Ig genes (40, 41). Pre-existing immunity in adults makes the characterization of the responses after seasonal vaccination challenging, so the 2009 H1N1 pandemic (pH1N1) influenza virus was a great opportunity to better understand the immune responses to influenza. Indeed, the pH1N1 HA was remarkably divergent from the HAs of the seasonal vaccines (even with a stem region quite conserved). Surprisingly, the highest numbers of deaths during the 2009 H1N1 pandemic were registered among the younger population, while the older population showed pre-existing protective immunity. How to explain the unexpected level of deaths among adults that is typically the most resistant group to influenza infections? It was suggested that adults had too low frequencies of cross-specific B cells to generate protective levels of cross-neutralizing antibodies against HA (42). On the contrary, the older population (over 65 years old) showed a very low incidence of infection and hospitalization (42–45), probably due to their life-long accumulation of an expanded reservoir of stem-specific cross-reactive memory B cells that efficiently responded to the 2009 pH1N1 virus (42). In addition, a close antigenic relation was found between the HA of the 2009 pH1N1 virus and the HA of influenza viruses that had circulated before 1950; hence, neutralizing antibodies against the HA globular head may also have contributed to protect the elderly population (46, 47).

In 2010, Lanzavecchia et al. reported that some individuals who received the seasonal influenza vaccine developed cross-reactive antibodies able to neutralize viruses belonging to different HA subtypes (H1, H2, H5, H6, and H9), including the pH1N1 isolate. By immortalizing IgG-expressing B cells, Lanzavecchia et al. showed that heterosubtypic monoclonal antibodies bound to acid-sensitive epitopes in the HA stem region, used different VH genes and carried high frequency of somatic mutations (24, 48, 49). More recently, the same group showed that most of the HA stem-specific antibodies are characterized by the use of the heavy-chain variable-region VH1-69 gene, only few polymorphisms are functional, and that few single somatic mutations are sufficient to promote high-affinity HA-specific antibodies (50).

The above studies have enhanced our understanding of influenza-specific B cell responses, and helped to set the primary goals in the development of next-generation anti-influenza therapies and vaccines. A major objective is to promote the generation of HA-specific broadly neutralizing antibodies in order to target cross-protective epitopes that are present among multiple strains. A second objective is to promote long-lasting memory B cells and plasma cells, hopefully for the entire life. Several strategies are today evaluated to achieve such goals including the use of adjuvants in vaccine formulation, heterologous prime-boost strategies, and antigen design with a “minimalistic-approach.”

## Cutting-Edge Strategies for Inducing Protective Anti-Influenza Immune Responses

How to translate our knowledge of the influenza-specific humoral responses into novel strategies that specifically elicit the ideal protective immunity? As primary goals, successful vaccination strategies should confer cross-protection against multiple strains of influenza virus, and should boost long-lasting protective immunity in subjects with weakened immunity, as well as in younger and elderly populations.

A very promising strategy to meet those purposes is based on the use of particular adjuvant formulations. Adjuvants have been used in influenza vaccines for decades, usually in combination with split or subunit vaccines with the major goal to enhance their intrinsic immunogenicity (51). Although aluminum salts are potent adjuvants for most of the subunit antigens present in licensed vaccines, they seem not to be good adjuvants for influenza antigens. Instead, oil-in-water emulsions, like MF59, have been successfully used in influenza vaccines for the past 20 years with outstanding results (52, 53). MF59 not only induces high titers of influenza-specific antibodies but also cross-reactive responses against different clades of influenza viruses (54–56). Khurana et al. showed that MF59 adjuvant promotes high titers of HA-specific antibodies and expands the overall diversity of the influenza-specific antibody repertoire (14, 57). MF59 also promotes persistence of long-lasting memory B cells and increases the affinity of the antibody responses, not only in adults but also in younger and elderly (14, 52, 56–58); such evidences have shed light on the use of oil-in-water emulsion as adjuvants for influenza vaccines. Furthermore, oil-in-water adjuvants may prevent the effect of the “original antigenic sin” that is the propensity of the immune system to preferentially utilize immunological memory instead of inducing novel responses, hence limiting the development of an expanded B cell repertoire (59–61). Although their mechanisms of action are still not fully understood, MF59 and AS03 (62), the other oil-in-water adjuvant used for pandemic vaccines, represent an important tool on the way to develop broadly protective influenza vaccines. An increased risk of narcolepsy was found few years ago following vaccination with AS03-adjuvanted split influenza vaccine used in several European countries during the A/California/07/09 H1N1 influenza pandemic, but multiple subsequent studies have not confirmed any possible association between vaccination and narcolepsy (63–66).

An alternative strategy consists of heterologous prime/boost vaccinations. When the immune system encounters for the first time an influenza antigen, it generates specific antibodies and long-lasting memory B cells. Many influenza epitopes shift every year, so a second encounter with the antigen will recruit naïve B cells, which are specific for the new shifted epitopes and will also expand the pre-existing pool of memory B cells that is specific for the most conserved epitopes (30, 67, 68). Subsequent immunizations with divergent influenza antigens, the “prime/boost strategy” might expand the memory B cells specific for the most conserved epitopes that usually are under-represented in the B cell repertoire, hence inducing cross-protective immunity. This approach has been shown to be successful by Wang et al. who used a gene-based heterologous prime/boost strategy to induce cross-protection. Mice were sequentially immunized with DNA coding for the HA of different influenza A H3 virus strains (A/Hong Kong/1/1968, A/Alabama/1/1981 or A/Beijing/47/1992) and boosted with another H3 virus, A/Wyoming/3/2003; mice developed cross-neutralizing antibodies and protective capacity against multiple subtypes of H3 viruses (69). In a similar study, Wei et al. immunized mice twice with the same HA strains, but using a different delivery system for priming and for boosting. Mice primed with a DNA plasmid encoding H1N1 HA or H3N2 HA from the 2006/2007 vaccine strains and boosted with the trivalent 2006/2007 seasonal vaccine, developed enhanced neutralizing antibodies against diverse H1N1 strains compared to mice receiving only DNA or seasonal vaccine and showed higher levels of protection after infection (18). The above studies provided the proof-of-concept that a prime/boost strategy can increase the production of broadly neutralizing antibodies, and suggested that a combined strategy involving nucleic acids/proteins may have the benefit of expanding the antibody repertoire as well as inducing a different type of cellular immunity (18, 70). We further speculate that sequential immunizations with different HA proteins properly formulated with oil-in-water emulsion adjuvants (MF59 or AS03), may truly maximize the broadly neutralizing repertoire against influenza compared to non-adjuvanted vaccine formulations.

The minimalist approach is an innovative strategy that is evaluated today to promote cross-protective humoral responses. It is based on the design of antigens composed only of cross-protective epitopes, in order to focus the immune system on the desired response and generate cross-protective immunity. This approach is strongly supported by the fact that most of the broadly neutralizing antibodies identified until today are directed against the stem region of HA, and very few against its globular head (17, 23, 24, 26, 28). The minimalistic approach for antigen design has demonstrated to be successful in mice immunized with a “headless” HA, an antigen composed by the complete HA2 polypeptide and some regions of HA1 that both form the stem part of HA. Such antigen maintained the structural integrity of the conserved stem domain, but lacked the globular head with its immunodominant strain-specific epitopes (71). Sera form mice receiving the “headless HA” showed broader reactivity against heterologous strains than sera from mice vaccinated with the full-length HA and were protected against lethal virus challenge. Similar findings were obtained by using a stabilized HA2 peptide (72). Furthermore, Wang et al. designed a 60-amino-acid peptide to reproduce a long α-helix (LAH) of HA2 recognized by a broadly neutralizing monoclonal antibody, the clone 12D1 (73). The LAH peptide was not much immunogenic by itself, but when coupled to a carrier protein (KLH)-induced protection in mice challenged with divergent subtypes of influenza viruses, including H3N2, H5N1, and H1N1 strains; this work represents the most important proof that a carefully designed immunogen can be used in influenza vaccines to skew the B cell responses toward the epitope of interests. However, some concerns have been raised regarding the development of a vaccine to elicit HA2 stem-targeting antibodies, not only because the stem region is poorly immunogenic by itself (requiring further optimization of the formulation with adjuvants or protein carriers) but also, in some circumstances, anti-stem antibodies have been observed to be detrimental for the host. Indeed, in a swine experimental model, Khurana et al. showed that a vaccine inducing anti-stem antibodies may have the risk to worsen the outcome of the influenza infection (74, 75).

Other groups have characterized cross-protective epitopes included in the HA globular head; in particular, Whittle et al. have identified a broadly neutralizing antibody that recognizes the receptor-binding pocket of HA and have suggested that a modified HA globular head could be used for epitope-based antigen design to promote broadly neutralizing antibodies (76, 77).

Although not strictly related to the “minimalistic approach” for antigen design, some work recently published by Giles et al. described a new computationally optimized broadly reactive antigen (COBRA) based on the structure of the HA from H5N1 subtype; mice and non-human primates immunized with this antigen develop broadly reactive antibodies and are protected from H5N1 challenge (78–80).

## Conclusion

Current influenza vaccines confer limited cross-protection against different strains of influenza and often fail to promote protective immunity in high-risk populations. Scientists are today evaluating multiple strategies to develop a universal influenza vaccine able to confer cross-protection, long-lasting immunity, and to be effective in subjects with weakened immunity. Such strategies include the use of oil-in-water emulsion adjuvants, heterologous prime/boost strategies, and antigen design. All these new strategies aim at inducing influenza-specific neutralizing antibodies that would confer sterilizing immunity in vaccinated hosts, and HA is the ideal antigen candidate to meet this purpose. Some groups are also evaluating alternative antigen candidates, such as NA, NP and M2, which are well conserved in multiple influenza strains and generate protective immunity through non-neutralizing antibodies helping to control the infection; hence, a multi-component vaccine not limited to HA antigen can be also considered. Each of the above strategies is promising to be successful, and most likely a combination of them will provide a universal influenza treatment in the future.

## Conflict of Interest Statement

Oretta Finco, Giuseppe Del Giudice and Sylvie Bertholet are employees of Novartis Vaccines and Diagnostics S.r.l. (a GSK company). Giuseppe Lofano and Arun Kumar have no conflict of interest to declare.
